# Cytosine 5-hydroxymethylation regulates VHL gene expression in renal clear cell carcinoma

**DOI:** 10.18632/oncotarget.19070

**Published:** 2017-07-07

**Authors:** En-Guang Ma, Yu-Feng Bai, Wei Cao, Yan Cao, Yong-Gang Huang, Huan-Chen Cheng, Rui-Hua An

**Affiliations:** ^1^ The Department of Urology, The First Affiliated Hospital of Harbin Medical University, Harbin, China; ^2^ The Department of Urology Surgery, The First Hospital of Harbin, Harbin, China; ^3^ The Department of Urinary Surgery, The Second Affiliated Hospital of Harbin Medical University, Harbin, China; ^4^ The Department of Urology, Harbin Medical University Cancer Hospital, Harbin, China; ^5^ Institute of Harbin Hematology & Oncology, The First Hospital of Harbin, Harbin, China

**Keywords:** DNA methylation, hydroxymethylation, VHL, renal clear cell carcinoma

## Abstract

Cytosine5-hyxymethylation (5hmC)which is a new epigenetic modification form plays important roles in the development and progression of tumors. In the present study, we observed that levels of 5hmC in the promoter region of Von Hippel-Lindau (VHL) were lower in 97 samples of renal clear cell carcinoma tissue than in matched adjacent benign tissues. Moreover, when the cancer tissue samples were divided based on pathological staging, VHL expression and the level of 5hmC in the VHL promoter were both lower in pathological grade III tumors than in grades I or II. Correspondingly, expression of TET1, which catalyzes the formation of 5hmC, was also lower in grade III renal clear cell carcinomas than in grade I or II disease. These findings suggest the 5hmC level on VHL is a key determinant of the gene's expression and may participate in the occurrence and development of renal clear cell carcinoma. Thus the 5hmC level may be a useful indicator for early diagnosis and appropriate treatment of renal clear cell carcinoma.

## INTRODUCTION

Renal clear-cell carcinoma (RCC) is a renal cortical tumor characterized by malignant cells with clear cytoplasm. RCCs account for 75% of kidney cancers [1], and the incidence of RCC has been increasing in recent years. Treatment for RCC is dominated by surgery. The curative effects of radiotherapy and chemotherapy, and thus the long-term prognosis after treatment, are poor [2]. Both surgical treatment and chemotherapy were closely related to disease grade. Consequently, early diagnosis, staging and treatment of RCC are crucial.

Methylation of cytosine to 5-methycytosine (5mC) is a well-studied epigenetic mechanism of gene regulation [3]. Changes in 5mC can be detected early during tumor-igenesis, and 5mC is involved in both the development and progression of tumors [4]. The ten-eleven-translocation (TET) family of enzymes are able to convert 5mC to 5-hydroxymethylcytosine (5hmC), which facilitates gene expression [5]. This newly identified epigenetic modification is mainly distributed at gene transcription enhancer and insulator binding sites [6]. Kraus et al [7] reported that 5hmC levels were significantly lower in brain tumor tissues than normal brain tissues. Orr et al [8] showed that reductions in 5hmC levels are related to the degree of tumor malignancy in nerve glioma. In addition, Thomson et al [9] showed that changes in 5hmC levels occur earlier than changes in 5mC, and that the changes are very stable. This suggests the 5hmC level is a potential marker for early tumor diagnosis and may be predictive of prognosis. But although there have been a number of studies comparing 5hmC levels between tumors and control tissues, only Wielscherc et al has demonstrated changes 5hmC on a single gene (LZTS1) in cancer (breast cancer) [10].

Von Hippel-Lindau (VHL) is a tumor suppressor gene involved in oxygen and energy-dependent promotion of protein ubiquitination and proteosomal degradation [11]. In humans, VHL gene is located on chromosome 3q25.26 and includes two isoforms: pVHL19 and pVHL30. The tumor suppressor effect of VHL protein disappeared when the two alleles of VHL gene were mutated or inactivated, leading to the occurrence of RCC [12]. VHL gene, whose product inhibits induction of tumor cell proliferation by Jade-1 or HIF-1, is the most studied gene in RCC [13]. Lutz et al [14] reported that inactivation of VHL expression influenced the polarization of tubular epithelial cells and the formation of primary cilia, which led to the occurrence of RCC. In that context, and taking into consideration that 5hmC and 5mC levels within the genome are usually maintained in relative balance [15], we investigated 5mC and 5hmC levels on VHL gene as a potential marker for diagnosis, staging and treatment of RCC.

## RESULTS

### Analysis of VHL expression and 5mC in RCC and adjacent benign tissues

**Figure 1:**
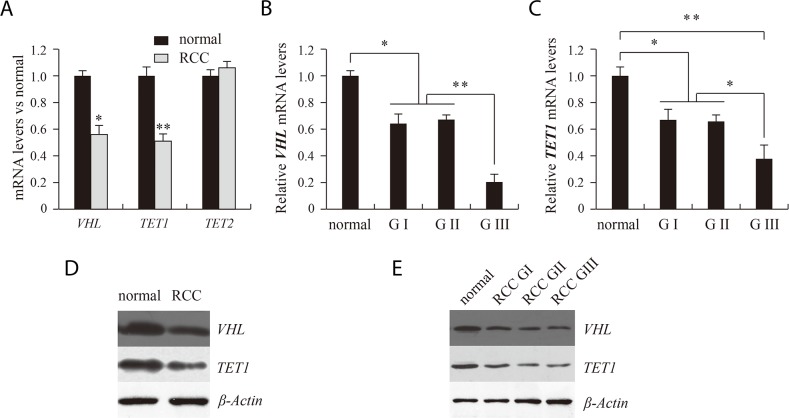
Analysis of VHL, TET1 and TET2 expression using real time PCR **(A, B, C)** and western blotting **(D, E)**. RCC and normal indicate renal clear cell carcinoma and matched adjacent benign tissues. G indicates the pathological grade of RCC. *P<0.05, **P<0.01 vs. normal. There were corresponding differences in expression of VHL and TET1 mRNA and protein between normal and RCC tissue **(D)** and between the different pathological grades of RCC **(E)**.

Real-time PCR results showed that levels of VHL mRNA were significantly lower in RCC tissue than in matched adjacent benign tissue (Figure 1A). Correspondingly, Western blot results showed that levels of VHL protein were also significantly lower in RCC than adjacent benign tissues (Figure 1D). When the RCC tissues were divided into different pathological grades, no difference in the levels of VHL mRNA or protein were detected between grades I and II. By contrast, but levels of both VHL mRNA and protein were significantly reduced in grade III (Figure 1B and 1E)

**Figure 2:**
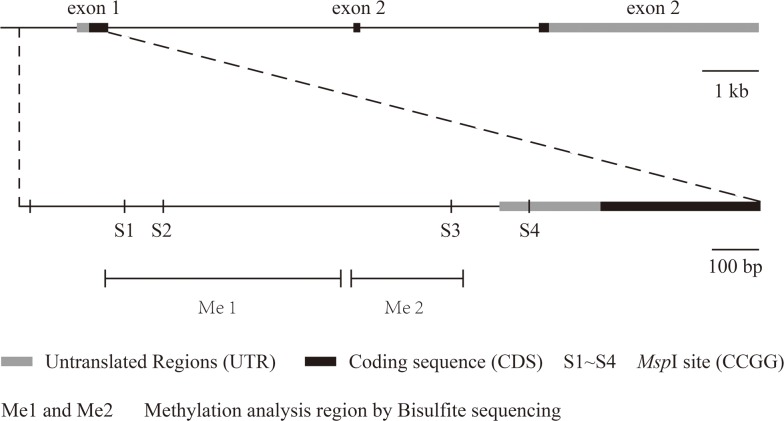
The structure of the VHL gene and its detected 5hmC sites

**Figure 3:**
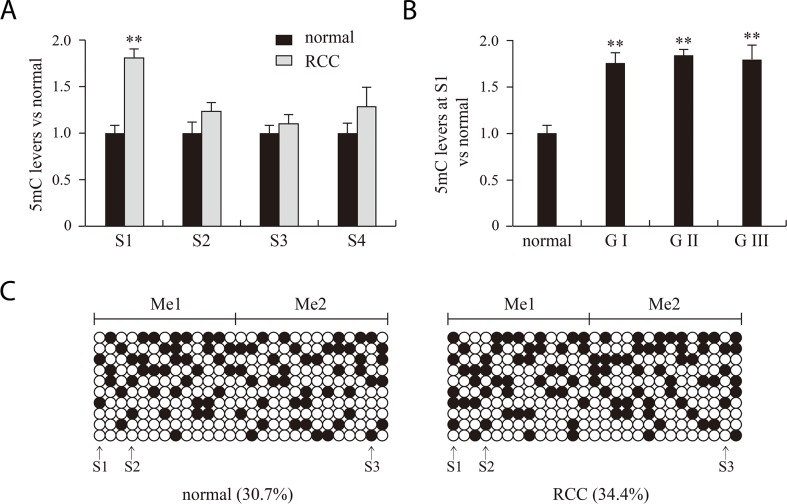
5mC analysis of normal, RCC tissues and different RCC grades with bisulfite treatment combined with PCR products sequencing **(A, B)** and real time PCR **(C)** methods. RCC and normal indicate renal clear cell carcinoma and matched adjacent benign tissues. G indicates the pathological grade of RCC. **P<0.01 vs. normal. Me1 and Me2 indicate the two 5mC regions detected. Filled and open circles represent methylated and unmethylated CG dinucleotides, respectively. S1, S2, S3 and S4 indicate four *MSP*I sites (CCGG).

We chose four *Msp*I sites (S1, S2, S3 and S4) in the promoter and 5′ untranslated regions of VHL to analyze 5mC and 5hmC levels. Figure 2 presents a structure diagram of the VHL promoter region. The fragment used for PCR product sequencing includes 27 CG dinucleotides that cover three *Msp*I sites (S1, S2 and S3). The levels of 5mC at the 27 CG dinucleotides did not significantly differ between the RCC and matched adjacent benign tissues (Figure 3C). However, the real-time PCR results showed that 5mC at S1 was significantly higher in RCC tissues than adjacent benign tissues, but the difference was not significant at S2-S4. When we measured the 5mC levels at S1 in RCC tissues at different pathological grades, we detected no difference among the three pathological grades (Figure 3A and 3B).

### VHL 5hmC is significantly decreased in patients with grade III RCC

**Figure 4:**
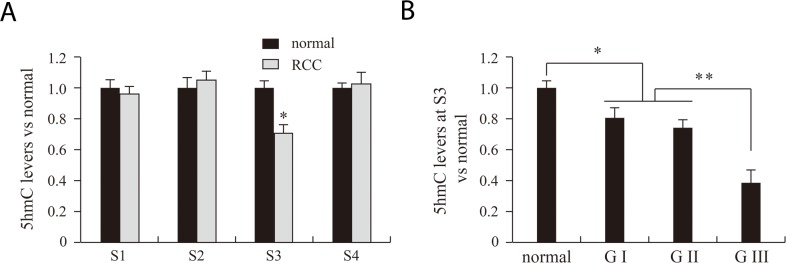
VHL 5hmC analysis of normal, RCC tissues and different RCC grades RCC and normal indicate renal clear cell carcinoma and matched adjacent benign tissues. S1, S2, S3 and S4 indicate the four *Msp*I sites (CCGG) used for 5hmC analysis. G indicates the pathological grade of RCC. *P<0.05, **P<0.01 vs. normal.

Although VHL expression was significantly lower pathological grade III RCC tissue than in grades I and II, the difference in 5mC levels did not significantly differ among the three grades. This suggests 5mC in our detected regions does not influence VHL expression. For that reason, we next examined levels of VHL 5hmC in RCC and adjacent benign tissues. Real-time PCR results indicated that 5hmC levels at S3 were significantly lower in RCC tissues than adjacent benign tissues (Figure 4A). Moreover, 5hmC at S3 was significantly lower in grade III RCC tissue than in grades I and II tissues (Figure 4B).

### TET1 could regulate VHL 5hmC

Ten-eleven translocation methylcytosine dioxygenase 1 (TET1) and TET2 are 5mC hydroxylases, which catalyze the conversion of 5mC into 5hmC. We therefore assessed expression of TET1 and TET2 to further study the mechanism of the 5hmC reduction RCC tissue. Real-time quantitative PCR results showed that TET1 mRNA levels were significantly lower in RCC than matched adjacent benign tissues, while TET2 expression did not differ between the two tissue types (Figure 1A). Moreover, as with VHL, TET1 transcription was significantly lower in pathological grade III RCC tissue than grades I and II (Figure 1C). Using western blotting, we detected corresponding differences in the levels of TET1 protein (Figure 1D and 1E).

## DISCUSSION

RCC originates from the renal parenchyma is one of the most common malignant tumors of the urinary system. Although there has been substantial progress in the diagnosis and treatment of RCC, the prognosis of RCC with distant metastasis remains poor [16]. It is therefore necessary to identify new molecular markers enabling the early diagnosis and treatment of RCC. Although 5hmC is reportedly important for regulating gene expression [17], there are no reports on RCC-related changes in 5hmC levels on single genes. In the present study, we found that expression levels of the tumor suppressor VHL are much lower in RCC tissues than adjacent benign tissues, and the expression is significantly lower in pathological grade III disease than in grades I and II. VHL may thus play a key role in the occurrence and development of RCC.

It is now well established that DNA methylation at the C5 position of the cytosine bases (5mC) is an epigenetic modification of the mammalian genome that is important in transcriptional regulation [3]. Hypermethylation of CpG islands within the promoter and 5′ regions of genes is an important epigenetic mechanism for suppressing gene expression [4]. In our study, no difference in 5mC levels was detected between RCC tissues and matched adjacent benign tissues. Although the 5mC level at S1 was significantly increased in RCC tissues, there was no obvious difference at the S2-S4 sites between RCC tissues and adjacent benign tissues. Moreover, the level of 5mC at S1 did not vary among Grades 1-II, though VHL expression was significantly decreased in grade III. This suggests other mechanisms are more strongly influence VHL expression.

TET1 and TET2 catalyze the transformation from 5mC to 5hmC and vice versa. Localization of 5hmC in regulatory regions such as transcription factor binding sites, promoters, and enhancers suggests that 5hmC has important regulatory functions [18, 19]. We found that like VHL expression, 5hmC levels were significantly decreased in RCC tissues and were reduced further in pathological grade III tissue than grades I and II tissue. This suggests that 5hmC, not 5mC, participates in the regulation of VHL expression. To explore that idea further, we assessed the expression of TET1 and TET2, which could activate VHL expression by converting 5mC to 5hmC in the gene's promoter region [20]. Our findings indicate that TET1 expression is significantly lower pathological grade III RCC tissue than in grades I and II, which corresponds to the levels of both 5hmC and VHL. This finding is consistent with earlier observations that TET1 is an essential tumor suppressor in prostate and breast cancers [21, 22]

In sum, our findings indicate that levels of 5hmC on VHL are lower in RCC tissues than matched adjacent benign tissues, and that the levels of VHL 5hmC are lower in pathological grade III RCC than grades I and II. By contrast, level of 5mC on VHL did not differ among tissues. It thus appears that the level of 5hmC on VHL regulates the gene's expression and may be participate in the occurrence and development of RCC.

## MATERIALS AND METHODS

### Patients and samples

A total of 97 frozen RCC tissue samples from patients with RCC and matched adjacent benign tissues were collected at the First Affiliated Hospital of Harbin Medical University and the Tumor Hospital of Heilongjiang Province between 2010 and 2015. Among the 97 RCC patients, the median age was 56. The clinical information for the patients is listed in Table 1. All patients provided informed consent for the experimental use of the surgical samples. The tissue specimens were snap frozen and stored at −80°C until used for experiments.

**Table 1:** Clinical features of renal clear cell carcinoma

Characteristics	Patients (n =97)
Gender	
Female	n = 45 (46%)
Male	n = 52 (54%)
Age	
≤ 50	n = 4 (4%)
50 ∼ 60	n = 63 (65%)
60 ∼ 70	n = 22 (23%)
≥ 70	n = 8 (8%)
Fuhrman grade	
Low: 1	n = 28 (29%)
Low: 2	n = 34 (35%)
High: 3	n = 35 (36%)
High: 4	n = 0 (0%)
Tumor location	
Left	n = 51 (53%)
Right	n = 46 (47%)
Tumor size	
> 7cm	n = 56 (58%)
≤ 7cm	n = 41 (42%)
Tumor metastasis	n = 35 (36%)
Tumor necrosis	n = 24 (25%)
Collecting system invasion	n = 21 (22%)
Perirenal fat invasion	n = 28 (30%)
Sinus fat invasion	n = 4 (4%)
Renal vein invasion	n = 45 (46%)

### DNA extraction and RNA isolation

Approximately 40 mg each of RCC tissue and adjacent benign tissue were separately ground and divided into two equal parts for extraction of DNA and RNA. Genomic DNA was extracted using a DNA Extraction Kit (Tiangen, Beijing, China) according to the kit's specifications. Total RNA was isolated using TRIzol reagent (Invitrogen) according to the manufacturer's instructions. The RNA samples were treated with Amplification Grade DNase I (TaKaRa, Tokyo, Japan) for 60 min at room temperature. First strand cDNA was synthesized using a RevertAid™ First Strand cDNA Synthesis Kit (TaKaRa).

### Detection of 5-hmC

DNA used for 5hmC analysis was first glycosylated using a Quest 5-hmC Detection Kit™ (Zymo Research, Irvine, CA) according to the manufacturer's instructions. Thereafter, glucose-5hmC-sensitive restriction endo-nucleases were able to cut the C and 5mC sites, but not the 5hmC sites. As a result, VHL 5hmC could be detected with real-time PCR using the treated DNA as a template. We used the ΔΔCt values (glucosylated-unglucosylated) to assess the 5hmC levels in the RCC and control tissues for all PCR fragments tested.

### Real-time PCR and western blot analysis

To detect 5hmC expression using real-time PCR, primers were designed based on the 5hmC sites in the VHL genomic sequence or the sequence of VHL mRNA. All primers are listed in Table 2. Real-time PCR was performed using an ABI 7500 system with SYBR®Premix Ex Taq™ (Takara) according to the manufacturer's instructions. The housekeeping gene GAPDH was used as internal control to evaluate relative expression levels.

**Table 2:** PCR products, primer sequences and PCR efficiency

PCR product	Sample	Left primer	Right primer	Size (bp)	R^2^	PCR efficiency
VHL S1	DNA	cttgtgatcagcccacttcagc	Gtcatgtttcctgccttcactg	144	0.998	98.82
VHL S2	DNA	atgacgcttttattgaagtgcag	tattaaggccctactatgtaccac	102	0.997	97.16
VHL S3	DNA	cgcctacagtaccaactactcg	tgagacagggtctcactctgtc	129	0.999	99.28
VHL S4	DNA	tacagtaacgagttggcctagc	gctcggtagaggatggaacg	126	0.990	107.13
VHL Me1-O	DNA	aggttttattatgttgttaggttgg	aattacaaaccttaaccactatacc	566	-	-
VHL Me1-I	DNA	aaagtattgggattataggtatgag	accttaaccactatacctaataaac	499	-	-
VHL Me2-O	DNA	tttgtaatttttgtattttgagagg	tttttaaaacaaaatctcactctatc	268	-	-
VHL Me2-I	DNA	taggaggattatttgaatttaggag	tttttaaaacaaaatctcactctatc	237	-	-
Control	DNA	gctctgcccatagatgcctttg	tccctggttttgacctggggga	91	0.999	102.52
VHL	mRNA	tctctcaatgttgacggacagc	gatcttcgtagagcgacctgac	148	0.992	99.13
TET1	mRNA	gcacataagataagggcagtgg	acttcaggttgcacggtctcag	137	0.992	101.19
TET2	mRNA	taggacatgatccaggaagagc	caggaatggacttagtctgttgc	142	0.996	98.86
GAPDH	mRNA	aaggtgaaggtcggagtcaac	tgaaggggtcattgatggcaac	106	0.998	99.37

R^2^ is the correlation coefficient. VHL Me1/2-O and VHL Me1/2-I are the outer and inner primers in methylation analysis of the region “Me1/2” through nested PCR. VHL S1, S2, S3 and S4 indicate the four *Msp*I sites (CCGG) used for 5hmC analysis.

Normal and RCC tissues were ground, and the total protein was isolated in lysis buffer and PMSF. Aliquots of protein were separated on SDS-PAGE, transferred to a PVDF membrane and detected using the anti-VHL, anti-TET1 and anti-β-actin antibodies (Cell signaling technology, Boston, MA).

### Analysis of 5mC

All samples for 5mC analysis were genomic DNA derived from RCC tissues and matched adjacent benign tissues. Genomic DNA was treated with a BisulFlash DNA Modification Kit (Zymo Research, Irvine, CA) before DNA methylation analysis. The treatment causes unmethylated cytosine to be converted to thymine, but the methylated cytosine remained unchanged. Real-time PCR and sequencing of the PCR products were then used for 5mC analysis. The real-time PCR method was similar to that used for 5hmC analysis. The PCR product sequencing enabled detection of CG dinucleotides and verified the accuracy of the real-time PCR method. To amplify CG dinucleotides, primers were designing primers based on the sequences after treatment. Thereafter, the PCR products were subjected to T-A cloning, and 10 positive clones were chosen for sequencing. The 5mC analysis entailed assessing the difference in CG dinucleotides between the original sequence and the sequencing results.

### Statistical analysis

All data are presented as the average of three independent experiments. The 2^−△△Ct^ method was used to calculate the relative expression and the 5mC and 5hmC levels in each gene. Statistical analysis was done using unpaired Student's t test. Values of p <0.05 and <0.01 were considered significant.
